# Effect of the Functionalization of Porous Silicon/WO_3_ Nanorods with Pd Nanoparticles and Their Enhanced NO_2_-Sensing Performance at Room Temperature

**DOI:** 10.3390/ma11050764

**Published:** 2018-05-10

**Authors:** Xiaoyong Qiang, Ming Hu, Boshuo Zhao, Yue Qin, Ran Yang, Liwei Zhou, Yuxiang Qin

**Affiliations:** 1 School of Microelectronics, Tianjin University, Tianjin 300072, China; shawn_q@tju.edu.cn (X.Q.); huming@tju.edu.cn (M.H.); 2200_lkj@sina.com (B.Z.); gkintju@tju.edu.cn (Y.Q.); yangran111998@126.com (R.Y.); zlw19890608@163.com (L.Z.); 2 Key Laboratory for Advanced Ceramics and Machining Technology, Ministry of Education, School of Materials Science and Engineering, Tianjin University, Tianjin 300072, China

**Keywords:** room temperature, gas sensor, porous silicon, Pd nanoparticles, WO_3_ nanorods

## Abstract

The decoration of noble metal nanoparticles (NPs) on the surface of metal oxide semiconductors to enhance material characteristics and gas-sensing performance has recently attracted increasing attention from researchers worldwide. Here, we have synthesized porous silicon (PS)/WO_3_ nanorods (NRs) functionalized with Pd NPs to enhance NO_2_ gas-sensing performance. PS was first prepared using electrochemical methods and worked as a substrate. WO_3_ NRs were synthesized by thermally oxidizing W film on the PS substrate. Pd NPs were decorated on the surface of WO_3_ NRs via in-situ reduction of the Pd complex solution by using Pluronic P123 as the reducing agent. The gas-sensing characteristics were tested at different gas concentrations and different temperatures ranging from room temperature to 200 °C. Results revealed that, compared with bare PS/WO_3_ NRs and Si/WO_3_ NRs functionalized with Pd NPs, the Pd-decorated PS/WO_3_ NRs exhibited higher and quicker responses to NO_2_, with a detection concentration as low as 0.25 ppm and a maximum response at room temperature. The gas-sensing mechanism was also investigated and is discussed in detail. The high surface area to volume ratio of PS and the reaction-absorption mechanism can be explained the enhanced sensing performance.

## 1. Introduction

Rapid development of global urbanization and industrialization has caused serious air pollution and endangered the natural environment and human health, therefore, detection of hazardous and harmful gases is of great need [1]. Nitrogen dioxide (NO_2_), as one of the main air pollutants, has caused the greatest concern, as it leads to smog and acid rain in metropolitan areas, as well as ozone formation in the lower atmosphere [2]. The safety standard for NO_2_ in the air is 3 ppm, as suggested by the American Conference of Governmental Industrial Hygienists [3]. Hence, a low cost, highly sensitive, selective, and reliable gas sensor is required to monitor NO_2_ leakage. There is an urgent demand for developing high-quality gas-sensitive materials accordingly. Among the various gas-sensitive materials, resistive-type nanostructured metal oxide semiconductors (MOS), such as TiO_2_, In_2_O_3_, SnO_2_, ZnO, CuO, and WO_3_ [4,5,6,7,8,9] have been most attractive in terms ofNO_2_ detection because of their high sensitivity, simplicity, and low cost [10]. WO_3_, as a typical n-type semiconductor with stable physicochemical properties, is promising for the detection of NO_2_ in recent years due to its excellent sensitivity and selectivity to NO_2_ compared with other gases. The first study to use WO_3_ as a sensing material revealed that WO_3_ is highly sensitive to NO_X_, but relatively insensitive to some reducing gases, such as CO, H_2_, and NH_3_ [11].

However, resistive-type MOS gas sensors are generally poor selectively to many gases because many gas species may cause simultaneous resistance changes, making it impossible to properly identify the gas with a single sensor [12]. Several approaches, such as structure manipulation, decoration of noble metals (including Au, Pt, and Pd [13,14,15]), and heterostructure formation have been reported by several researchers to improve the selectivity of MOS gas sensors [16,17,18]. Among these approaches, noble metal nanoparticle (NP) decoration is promising in gas-sensing enhancement. For instance, Vuong et al. [14] decorated Au NPs on the surface of WO_3_ nanowires for response enhancement to H_2_S and CH_4_ gases. Yasuhiro Shimizu et al. [19] functionalized SnO_2_ thick film by Pd NPs for enhanced NOs gas-sensing performance. Phanichphant et al. [20] synthesized Pt-loaded WO_3_ nanoparticles to achieve a highly selective H_2_ gas sensor. The other challenge is that MOS gas sensors generally lack compatibility with integrated circuit devices, because integrated circuits are essentially built on silicon. MOS gas sensors have to be worked at different temperature ranges to distinguish between target gases; their working temperatures are usually above 150 °C [21,22,23], which limits their future applications due to high energy consumption.

Porous silicon (PS) is a nanostructured material prepared by chemical or electrochemical etching of crystalline silicon. It was accidentally discovered by Uhlir at Bell Laboratories in the mid-1950s [24]. The unique features of the PS—such as its extremely large surface area to volume ratio, its easy fabrication, its active surface chemistry, and its compatibility with microelectronic and MEMS (Micro-Electro-Mechanical Systems) inspired research into applications ranging from sensors to electronics, biomedicine, optics, solar cells, and batteries [25,26,27,28,29]. Barillaro et al. integrated a composite of PS/gold nanostructures into JFET (Junction Field-effect Transistor) to realize a novel chemitransistor gas sensor which shows a fast and reliable response to NO_2_ [30]. Tebizi-Tighilt et al. obtained a gas sensor based on PS and polypyrrole, which can operate at a low bias voltage. This indicates that it is advantageous for energy consumption [31]. Ibrahim designed an innovative PS-based sensor to identify alcohol which had relatively higher repeatability and easier positioning and flexibility in sensing [32]. It is realized that PS stands out as a preeminent platform for sensing applications. Composites consisting of PS and MOS have shown improved sensitivity and stability, even in low working temperatures. Some work about synthesizing MOS on PS substrate has been done to improve sensitivity and reduce the working temperature through thermal evaporation or hydrothermal methods. For instance, a tungsten oxide gas sensor based on PS substrate can detect NO_2_ at room temperature (RT, ~25 °C) [33,34] and is compatible with existing microfabrication techniques. Both noble metal NP decoration on MOS and composites consisting of PS and MOS can improve the gas-sensing performance (i.e., sensitivity, selectivity, stability, and working temperature) of MOS gas sensors. However, there are few papers studying MOS gas sensor decorating with noble metal NPs and combining these with PS substrate simultaneously to improve gas sensing performance.

In this work, we synthesized PS/WO_3_nanorods (NRs) functionalized with Pd NPs, investigated its gas-sensing properties at different temperatures (25–200 °C) under exposure to different gas concentrations (ranging from 0.25 to 2 ppm), and also examined the effect of Pd-loading amounts on response values and the response/recovery time of the PS/WO_3_ NRs. PS was prepared by galvanostatic electrochemical etching. PS/WO_3_ NRs were fabricated by thermal oxidizing W film, which was magnetron sputtered on the PS substrate. Pd NPs were decorated on the surface of WO_3_ NRs by in-situ reduction of the Pd complex, using the copolymer Pluronic P123 as a reduction agent and surfactant. Experimental results revealed that the NO_2_-sensing properties of the PS/WO_3_ NRs–Pd NPs were significantly enhanced by decorating Pd NPs and combining PS with WO_3_ NRs. The PS and Pd NPs complemented each other to realize enhanced gas sensitivity, better selectivity, and lower response temperatures. The maximum response was achieved at RT, which demonstrates that the PS/WO_3_ NRs–Pd NPs is of low power consumption and is promising for an RT resistive-type NO_2_ gas sensor.

## 2. Materials and Methods

### 2.1. Synthesis and Characterization of PS/WO_3_ NRs–Pd NPs

**Figure 1:**
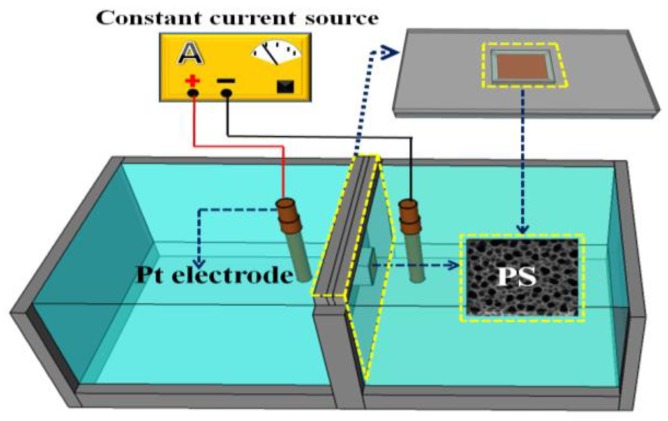
Schematic diagram of the Teflon double-tank cell.

PS was prepared using galvanostatic electrochemical etching methods in a Teflon double-tank cell. Figure 1 shows the schematic diagram of the Teflon double-tank cell. Before the electrochemical etching, p-type silicon wafers (Tianjin Institute of Semiconductor Technology, (100)-oriented, resistivity: 10–15 Ωcm, thickness: 400 ± 10 μm) were cut to a size of 24 × 9 mm, ultrasonically cleaned in acetone (Tianjin Kemiou Chemical Reagent Co., Ltd.), ethanol (Tianjin Kemiou Chemical Reagent Co., Ltd., Tianjin, China), and deionized water (Tianjin Kemiou Chemical Reagent Co., Ltd., Tianjin, China) successively for 15 min each, then immersed in the H_2_SO_4_ (AR, Tianjin Jiangtian Chemical Technology Co., Ltd.)/H_2_O_2_ (AR, 30 wt %, Tianjin Jiangtian Chemical Technology Co., Ltd.) (volume ratio 4:1) solution for 30 min to remove organic contaminants and dilute HF solution (Tianjin Kemiou Chemical Reagent Co., Ltd.) for 10 min to remove the oxide layer formed on the surface. After cleaning, the Si wafers were electrochemical etched in the electrolyte, which was comprised of 40 wt % hydrofluoric acid and 99.5 wt % *N,N*-dimethylformamide (DMF, Tianjin Jiangtian Chemical Technology Co., Ltd., AR, Tianjin, China) (volume ratio 1:3), with a current density of 60 mAcm^−2^ for 10 min at RT.

**Figure 2:**
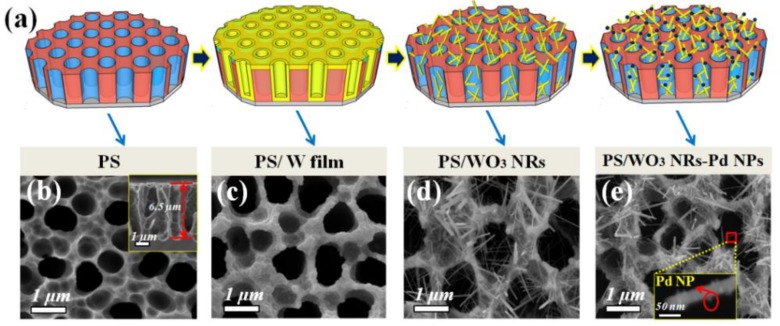
(**a**) Schematic illustration of the fabrication process of porous silicon(PS)/WO_3_ nanorods (NRs)–Pd nanoparticles (NPs); SEM images: top view of (**b**) PS (inset: cross-section view); (**c**) PS/W film; (**d**) PS/WO_3_ NRs; and (**e**) PS/WO_3_ NRs–Pd NPs (inset: magnification).

PS/W film was formed by DC magnetron sputtering (High Vacuum Multifunctional Magnetron Sputtering Coating Equipment SKY Technology Development Co., Ltd., Shenyang, China) a W target (Shanghai Qingyu Material Technology Co., Ltd., Shanghai, China, purity 99.999%) on the PS substrate. Before sputtering, the cavity pressure was pumped to 9 × 10^−5^ Pa, then 120 nm W film was deposited on the PS substrate directly after sputtering for 15 min with sputtering power of 110 W and cavity pressure of 2 Pa in a pure Ar (HexiDistirct, Tianjin six high-tech gas supply station, Tianjin, China, purity 99.999%) atmosphere. The PS/W film sample was thermally oxidized in a tube furnace (Hefei Kejing Materials Technology Co., Ltd., Hefei, China, GSL-1400X) for 1 h at 700 °C in an atmosphere of Ar and O_2_ (HexiDistirct, Tianjin six high-tech gas supply station, Tianjin, China) (volume ratio 30:0.5) to create PS/WO_3_ NRs. During the thermal oxidizingprocess, the vacuum of the tube furnace was kept at 1.5 torr. After cooling the PS/WO_3_ NRs samples to RT, Pd NPs were decorated on the surface of WO_3_ NRs via the in-situ reduction of a Pd complex at RT. During the reduction process, copolymer Pluronic P123 (Energy Chemical) was used as reduction agent and surfactant, without any other linker [35], and with the assistance of photochemical reduction [36]. The Pd complex was prepared by dissolving PdCl_2_ (Tianjin Guangfu Fine Chemical Research Insitute) in an aqueous NaCl (Tianjin Guangfu Fine Chemical Research Insitute, Tianjin, China) solution to obtain a Na_2_(PdCl_4_) solution. To investigate the influence of the amount of Pd decoration onNO_2_-sensing performance, three different quantities of PdCl_2_ (20, 40, and 60mg) were dissolved to form the Na_2_(PdCl_4_) solution. The final as-fabricated PS/WO_3_ NRs–Pd NPs samples are noted as PS/WO_3_–Pd20, PS/WO_3_–Pd40 and PS/WO_3_–Pd60, respectively. PS/WO_3_ NRs samples were immersed in the Na_2_(PdCl_4_) solution and magnetically stirred for 5 min. Then, a solution of 1.5 g Pluronic P123 dissolved in 40 mL H_2_O was added to the Na_2_(PdCl_4_) solution to reduce Pd^2+^ to Pd NPs. The reduction process was performed at RT for 7 h in atmospheric gas. After being collected and washed with deionized water and ethanol, the as-fabricated PS/WO_3_ NRs–Pd NPs samples were dried at 75 °C for 1 h in an oven to vaporize the surplus Na_2_(PdCl_4_) solution. Figure 2a shows the schematic illustration of the fabrication process of PS/WO_3_ NRs–Pd NPs. To explore the role of the PS substrate in gas-sensing performance, WO_3_ NRs decorated with Pd NPs and grown on a Si substrate were also prepared. These were named Si/WO_3_–Pd20. The whole fabrication process of Si/WO_3_ NRs–Pd NPs was similar to that of PS/WO_3_–Pd20, except using a Si substrate instead of a PS substrate.

Characterization of the obtained samples were performed using several techniques, such as field emission scanning electron microscopy (FESEM, ZEISS MERLIN compact, ZEISS, Jena, Germany), field emission transmission electron microscopy (FETEM, Tecnai G2 F20, Ames Laboratory Sensitive Instrument Facility, Washington DC, USA), and X-ray diffraction (XRD, RIGAKU D/MAX-2500 V/PC, RIGAKU, Tokyo, Japan, Cu Ka radiation).

### 2.2. Gas-Sensing Characterizations

The restive-type MOS gas sensors were fabricated via RF (Radio Frequency) magnetron sputtering two Pt electrodes (3 × 3 mm) on the top surface of the PS/WO_3_ NRs–Pd NPs samples, with the help of a shadow mask. The gas-sensing properties were assessed by measuring the resistance change of the gas sensors under exposure to the tested gases in a homemade static gas-sensing testing system [37]. During the testing process, NO_2_ in dried air was injected directly into the testing chamber with the desired concentration. The resistance of the PS/WO_3_ NRs–Pd NPs gas sensor was recorded successively. A calibrated heater was mounted on the backside of the sensor to adjust the working temperature. The ambient relative humidity was constant and kept at 40%.

The gas response is defined to be R_a_/R_g_ for NO_2_ gas and R_g_/R_a_ for the reducing gas, where R_a_ and R_g_ are the resistances of the gas sensor in the gas atmosphere and in the tested gas, respectively. The response time is defined as the time taken for 90% of the total resistance change to occur. Conversely, the recovery time is the time taken for 90% recovery of the resistance change. In order to ensure the reliability of the results, the gas-sensing performance of every gas sensor was tested repeatedly.

## 3. Results and Discussion

### 3.1. Materials Characterization

Figure 2b–e show the low-magnification SEM images of PS, PS/W film, PS/WO_3_ NRs, and PS/WO_3_ NRs–Pd NPs corresponding to the illustrated diagram (Figure 2a). As shown in Figure 2b, uniformly distributed pores with an average diameter of 0.93 μm and depth of 6.5 μm can be observed from the plane view and the cross-section view. Such pore sizes are beneficial to the growth of WO_3_ NRs inside the PS hole and the easy absorption–desorption of the tested gas. The porosity of the PS is about 40%. The determination of porosity is normally defined by gravimetric methods according to the following equation [38]:

(1) $$p\;\left(\%\right)=\frac{m_{1}-m_{2}}{m_{1}-m_{3}}$$

where m_1_ is the weight of the Si wafer before electrochemical etching, m_2_ is the weight after electrochemical etching, and m_3_ is the weight after removal of the porous layer in a 1 wt % KOH solution.

**Figure 3:**
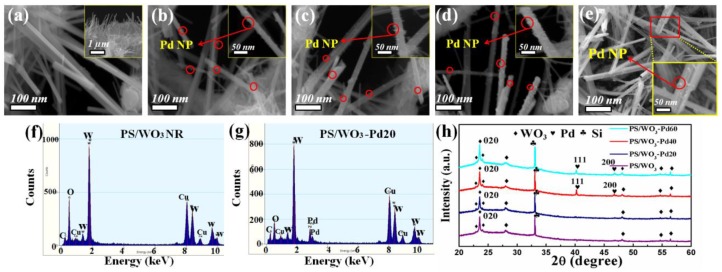
High-magnification SEM images of (**a**) PS/WO_3_ NRs: top view (inset: cross-section view); (**b**) PS/WO_3–_Pd20 (inset: single PS/WO_3_ NR–Pd20); (**c**) PS/WO_3–_Pd40 (inset: single PS/WO_3_ NR–Pd40); (**d**) PS/WO_3–_Pd60 (inset: single PS/WO_3_ NR–Pd60); and (**e**) Si/WO_3–_Pd20; EDS analyses of (**f**) PS/WO_3_ NRs; and (**g**) PS/WO_3–_Pd20; (**h**) XRD patterns of PS/WO_3_ NRs; PS/WO_3–_Pd20, PS/WO_3–_Pd40, and PS/WO_3–_Pd60.

Figure 2c shows the morphology of the PS/W film. Nearly 120 nm W film was successfully deposited on the surface and hole wall of PS. The morphology of the PS/WO_3_ NRs is shown in Figure 2d and Figure 3a. The top view (Figure 2d) and cross-section view (inset of Figure 3a) images show that uniform and dense WO_3_ NRs were distributed layer-by-layer on the inside and outside of the PS holes. It also shows arodlike nanostructure with a diameter of 30–60 nm and length of 1–4 μm and shows that the surfaces of WO_3_ NRs were clean (Figure 3a). Figure 2e and Figure 3b–d show the morphologies of PS/WO_3_ NRs–Pd NPs, nanoscale Pd NPs were homogenously distributed and strongly decorated the surface of the WO_3_ NRs. WO_3_ NRs kept their rodlike shape after the decoration of Pd NPs, which means that the decoration process of Pd NPs did not hurt the physical structure of WO_3_ NRs. Strong binding between the surface of WO_3_ NRs and the Pd NPs generated the potential barrier at the WO_3_ contact points, thereby forming the depletion region that contributed to the response of the materials [39]. In the decorating process of Pd NPs, controlling the decorating time and rate by adjusting the Pd complex concentration and the reduction time is important to preventing the agglomeration of Pd NPs, which lowers the sensing performance of the gas sensor. In this work, we varied the Pd NPs decorating amount by adjusting the concentration of the Pd complex solution. Figure 3b–d reveal that Pd NPs were decorated successively on the surface of WO_3_ NRs in all three samples, but the decorating amounts of Pd NPs increased obviously with the increase of the PdCl_2_ solution concentration. When adjusting the quantity of PdCl_2_ to 20 mg, Pd NPs distributed evenly on the surface of WO_3_ NRs and there was no Pd NPs agglomeration (Figure 3b). When the quantity of PdCl_2_ increased to 40 mg, there was much more Pd decoration on the WO_3_ NRs; the distribution density of Pd NPs increased and distribution spacing of Pd NPs decreased (Figure 3c). After the quantity of PdCl_2_was further increased to 60 mg, very few Pd NPs agglomerated and formed a bigger Pd NP agglomeration (Figure 3d). Figure 3e shows the morphology of Si/WO_3_ NRs–Pd20. It also shows that oblique WO_3_ NRs are distributed in a disorderly manner on the surface of the Si substrate. The diameters of the WO_3_ NRs ranged from 30 to 60 nm. Pd NPs successfully decorated the surface of WO_3_ NRs. Compared with PS/WO_3_ NRs–Pd20, the WO_3_ NRs that grew on a Si substrate showed higher density and cluster growth phenomenon. Figure 3f,g show the EDS spectra of PS/WO_3_ NRs and PS/WO_3_–Pd20. The O and W elements were from WO_3_ NRs, and the Pd element was from Pd NPs. The Cu and C elements can be attributed to the grids during the TEM testing process.

To investigate the crystal structure, typical XRD patterns of PS/WO_3_ NRs and PS/WO_3_ NRs–Pd NPs were carried out (Figure 3h). The main diffraction peaks of WO_3_ NRs are well indexed to the orthorhombic WO_3_ (JCPDS Card No. 71-0131).The (0 2 0) diffraction peak is the major peak, which indicates that the [0 2 0] direction was the preferential growth direction. The XRD patterns of PS/WO_3_–Pd40 and PS/WO_3_–Pd40 indicate the face-centered cubic crystal structure of Pd (JCPDS Card No. 46-1043). Reflection of Pd elements did not exist in the PS/WO_3_–Pd20 sample. This phenomenon may be attributed to the slight amount of Pd NPs decoration. It also can be observed that the intensity of the [1 1 1] diffraction peak of Pd is higher than that of the (2 0 0) diffraction peak in the Pd XRD patterns of PS/WO_3_–Pd40 and PS/WO_3_–Pd40, suggesting that the Pd NPs were highly crystalline and mainly bound by {1 1 1} facets [40].

**Figure 4:**
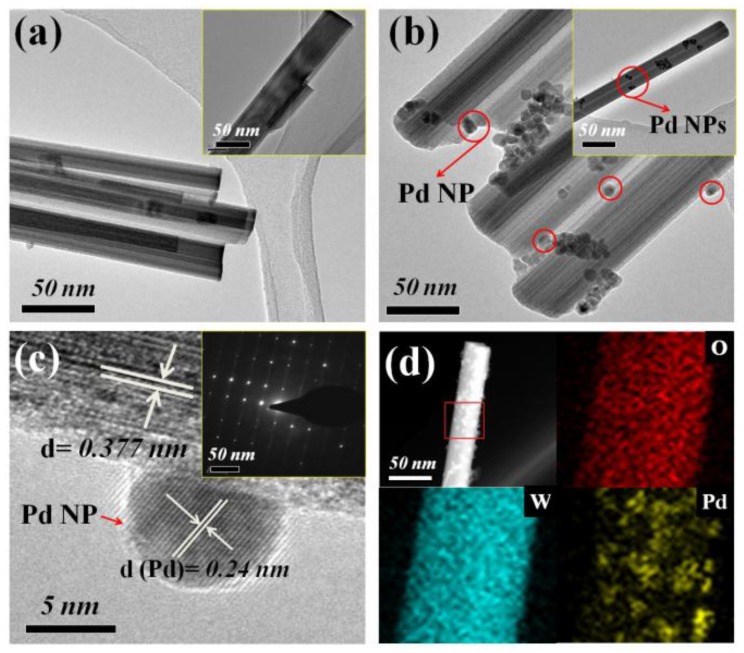
TEM images of (**a**) PS/WO_3_ NRs (inset: single WO_3_ NR); (**b**) PS/WO_3_–Pd60 (inset: single PS/WO_3_–Pd60); (**c**) HRTEM image of PS/WO_3_–Pd60 (inset: SAED image of WO_3_ NR); (**d**) STEM image and EDS mapping images of PS/WO_3_–Pd60.

Figure 4a,b show the TEM images of PS/WO_3_ NRs and PS/WO_3_–Pd60. As can be seen, the average diameter of WO_3_ NRs was about 40 nm (Figure 4a). After Pd decorating, WO_3_ NRs kept their rodlike structure and some Pd NPs agglomerated (Figure 4b), which is consistent with the SEM result in Figure 3d. Figure 4c shows the HRTEM image of PS/WO_3_–Pd60; the results reveal that spherical Pd NPs had an average diameter of 7.5 nm. The gaps between the lattice fringes of the Pd NPs and WO_3_ NRs were measured to be 0.24 and 0.377 nm, corresponding to the XRD results in Figure 3h. Some stacking faults were also observed in the WO_3_ NRs; these may be a number of defects, such as oxygen vacancies, which were induced in the crystal lattice during the growth process. The SAED (Selected Area Electron Diffraction) result further validates that the WO_3_ NR is a single crystalline. Few diffraction pots were not periodically distributed, indicating that there were two phases: WO_3_ and Pd. To further characterize the element distribution and decoration of Pd NPs, STEM imaging and EDS mapping of PS/WO_3_–Pd60 were performed, as shown in Figure 4d. The STEM image also demonstrates that Pd NPs were nearly uniformly decorated on the surface of WO_3_ NRs. Element analysis by EDS mapping reveals the existence of O, W, and Pd; O and W were originally from the WO_3_ NRs, whereas Pd was originally from the Pd NPs. The intensity of the O and W elements were obviously higher than that of the Pd element.

### 3.2. NO_2_-Sensing Performance

**Figure 5:**
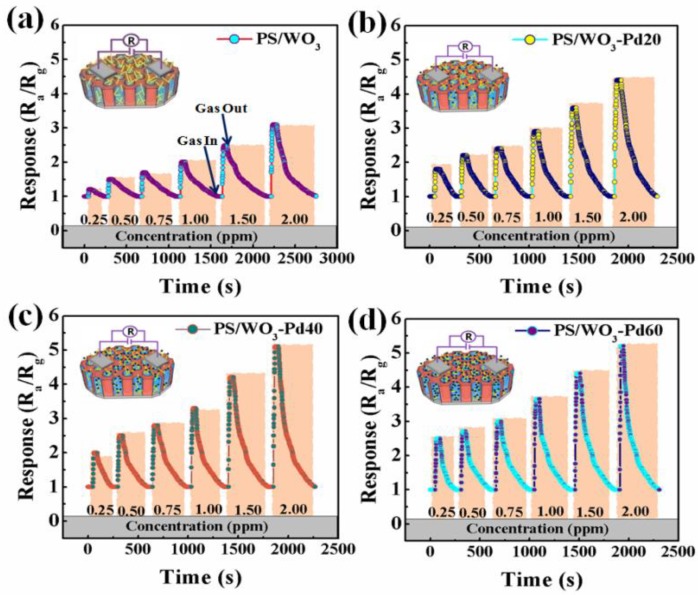
Dynamic gas response curves of (**a**) the PS/WO_3_ sensor; (**b**) PS/WO_3_–Pd20 sensor; (**c**) PS/WO_3_–Pd40 sensor; and (**d**) PS/WO_3_–Pd60 sensor to different concentrations of NO_2_ with varying times at RT.

The gas-sensing properties of the PS/WO_3_ NRs gas sensor and the PS/WO_3_ NRs–Pd NPs gas sensors were tested under exposure to various concentrations of tested gas at different temperatures. Figure 5a–d show the dynamic gas response curves of the PS/WO_3_ NRs sensor, PS/WO_3_–Pd20 sensor, PS/WO_3_–Pd40 sensor, and PS/WO_3_–Pd60 sensor under exposure to varying NO_2_ concentrations at RT. The response of the PS/WO_3_ NRs sensor, PS/WO_3_–Pd20 sensor, PS/WO_3_–Pd40 sensor, and PS/WO_3_–Pd60 sensor increased rapidly when exposed to NO_2_. They then recovered their initial value after being exposed to atmospheric gas, which means that the resistance of the four sensors decreased when in NO_2_ atmosphere, indicating p-type semiconductor characteristics. All four sensors exhibited a good response–recovery quality, which confirms stability and reversibility in practical applications. Compared with the PS/WO_3_ NRs sensor, the PS/WO_3_–Pd20 sensor, PS/WO_3_–Pd40 sensor, and PS/WO_3_–Pd60 sensor presented larger responses, validating that PS/WO_3_ NRs–Pd NPs sensors were more sensitive to NO_2_ than the PS/WO_3_ NRs sensor after decoration with Pd NPs. In Figure 5b–d, the responses of the PS/WO_3_–Pd20 sensor, PS/WO_3_–Pd40 sensor, and PS/WO_3_–Pd60 sensor to 0.25 ppm NO_2_ were around 2, indicating that PS/WO_3_ NRs–Pd NPs sensors are capable of NO_2_ detection down to a ppb level at RT, which has been a challenge in current environmental gas-sensing materials.

**Figure 6:**
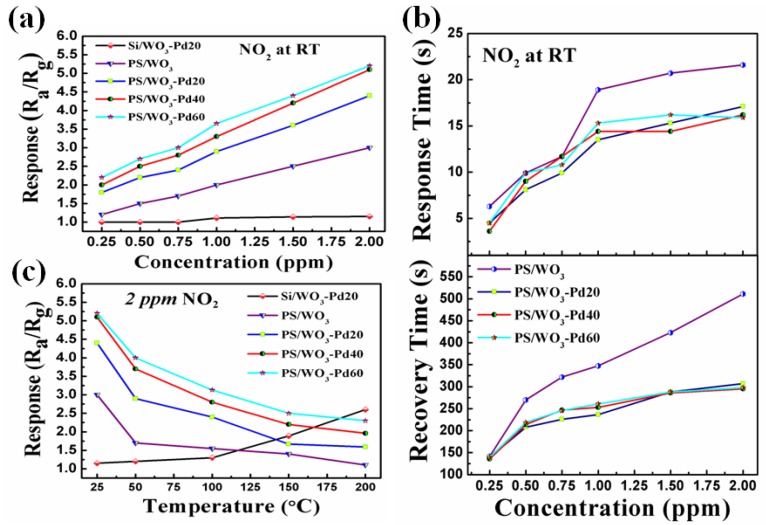
(**a**) Gas response of the Si/WO_3_–Pd20 sensor, PS/WO_3_ sensor, PS/WO_3_–Pd20 sensor, PS/WO_3_–Pd40 sensor, and PS/WO_3_–Pd60 sensor to 0.25, 0.5, 0.75, 1, 1.5, and 2 ppm NO_2_ at RT; (**b**) response–recovery time of the PS/WO_3_ sensor, PS/WO_3_–Pd20 sensor, PS/WO_3_–Pd40 sensor, and PS/WO_3_–Pd60 sensor to 0.25, 0.5, 0.75, 1, 1.5, and 2 ppm NO_2_ at RT; (**c**) the relationship between the response to 2 ppmNO_2_ and the operating temperature for the Si/WO_3_–Pd20 sensor, PS/WO_3_ sensor, PS/WO_3_–Pd20 sensor, PS/WO_3_–Pd40 sensor, and PS/WO_3_–Pd60 sensor.

Figure 6a shows the relationship between the sensor response, with concentrations of NO_2_ varying from 0.25 to 2 ppm at RT. The response of the Pd/WO_3_ sensor, PS/WO_3_–Pd20sensor, PS/WO_3_–Pd40 sensor, and PS/WO_3_–Pd60sensor increased obviously with the increase of the NO_2_ concentration. However, the response of the Si/WO_3_–Pd20 sensor had a small response and no change with increasing NO_2_, which indicates that, without the PS substrate, WO_3_–Pd20 can not detect such a low concentration of NO_2_ at RT. The decoration of Pd NPs dramatically enhanced the sensing performance of the PS/WO_3_ sensor. The response of the PS/WO_3_ sensor to 2 ppm NO_2_ was 3. The response of PS/WO_3_–Pd NPs sensors varied at 4.4, 5.1, and 5.2 when adjusting the quantity of PdCl_2_ from 20 mg to 40 mg, and then to 60 mg. The highest response was achieved by the PS/WO_3_–Pd60sensor to 2 ppm NO_2_, followed by the PS/WO_3_–Pd40 sensor, then the PS/WO_3_–Pd20 sensor. This result shows that a higher amount of Pd NPs decoration can result in a higher response. However, the sensor response increased only 2%, from 5 to 5.1, when the quantity of PdCl_2_ increased 50%, from 40 to 60 mg, whereas the sensor response increased 16% when the quantity of PdCl_2_ increased from 20 to 40 mg. This outcome is consistent with the observed SEM images in Figure 3b–d, where with the increase of PdCl_2_ quantity, Pd NPs tend to overlap or agglomerate, resulting in lowering the catalytic efficiency [41]. Overlapping or agglomerating of Pd NPs reduces the surface contact of WO_3_ NRs with oxygen molecules, leading to decreased capture of electrons from the conduction band of WO_3_ NRs. This thereby lowers the sensing performance [42]. Table 1 shows the comparisons of NO_2_ concentration, response, and working temperature of the PS/WO_3_–Pd60 sensor with those of other nanomaterial sensors. PS/WO_3_–Pd60 sensor achieved a higher response to a relatively low concentration of NO_2_ at RT compared with other nanomaterial sensors.

The response–recovery time is an important issue for a gas sensor in actual applications. Figure 6b shows the response–recovery time of the Pd/WO_3_ sensor, PS/WO_3_–Pd20 sensor, PS/WO_3_–Pd40 sensor, and PS/WO_3_–Pd60 sensor when increasing NO_2_ concentration from 0.25 to 2 ppm at RT. The response time of the PS/WO_3_ sensor was about 24 s and the recovery time was about 568 s under exposure to 2 ppm NO_2_, whereas the response time of PS/WO_3_–Pd20 sensor was about 10 s and the recovery time was about 339 s. The PS/WO_3_–Pd40 sensor and PS/WO_3_–Pd60 sensor nearly had the same response–recovery time as the PS/WO_3_–Pd20 sensor. All three PS/WO_3_ NRs–Pd NPs sensors had faster response–recovery times compared with the PS/WO_3_ sensor under exposure to the same NO_2_concentration, demonstrating that the decoration of Pd can reduce the response–recovery time.

Figure 6c shows the relationship between the response and operating temperature for the Si/WO_3_–Pd20 sensor, PS/WO_3_ sensor, PS/WO_3_–Pd20 sensor, PS/WO_3_–Pd40 sensor, and PS/WO_3_–Pd60 sensor. For the Si/WO_3_–Pd20 sensor, when the temperature was lower than 100 °C, there was almost no response. When the temperature was increased to 150 °C, the response was 1.89. When the temperature was further increased, the response increased accordingly. On the contrary, for the PS/WO_3_ sensor, PS/WO_3_–Pd20 sensor, PS/WO_3_–Pd40 sensor, and PS/WO_3_–Pd60 sensor, the response decreased with increasing operating temperatures, from RT to 200 °C. This phenomenon shows that PS plays a major role in lowering the working temperature due to its extremely large surface area to volume ratio and active surface chemistry. The optimum working temperature of a gas sensor is defined as the temperature in which the maximum response was achieved. Hence, the optimum working temperature of the PS/WO_3_ sensor, the PS/WO_3_–Pd20 sensor, the PS/WO_3_–Pd40 sensor, and the PS/WO_3_–Pd60 sensor was RT, which makes them promising low-consumption sensors.

**Figure 7:**
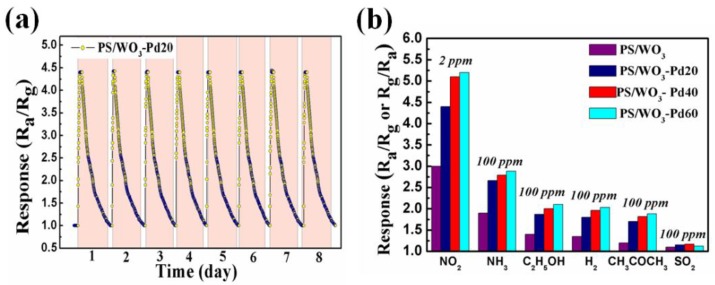
(**a**) The cyclic response curve of the PS/WO_3_–Pd20 sensor to 2 ppm NO_2_ at RT; (**b**) the response of the PS/WO_3_ sensor, PS/WO_3_–Pd20 sensor, PS/WO_3_–Pd40 sensor, and PS/WO_3_–Pd60 sensor to different gases at RT.

For practical applications, a gas sensor should have good stability and selectivity. Figure 7a shows the cyclic response curve of the PS/WO_3_–Pd20 sensor to 2 ppm NO_2_ at RT. The PS/WO_3_–Pd20 sensor exhibited nearly identical responses over 8 days of cyclic testing. Its good stability is thought to be highly related to the strong binding between WO_3_ NRs and Pd NPs. In order to study selectivity of the PS/WO_3_ sensor and PS/WO_3_ NRs–Pd NPs sensors, the gas responses to 100 ppm NH_3_, 100 ppm C_2_H_5_OH, 100 ppm H_2_, 100 ppm CH_3_COCH_3_, and 100 ppm SO_2_ were measured at RT (Figure 7b). The decoration of Pd NPs not only enhanced the gas response to NO_2_ but also enhanced the response to NH_3_, ethanol (C_2_H_5_OH), H_2_, acetone (CH_3_COCH_3_), and SO_2_. However, the response to NO_2_ can be detected in lower concentrations (0.25–2 ppm), but the response to NH_3_, C_2_H_5_OH, H_2_, CH_3_COCH_3_, and SO_2_ can only be detected when the concentration is increased to 100 ppm. Thus, we believe that the PS/WO_3_ NRs–Pd NPs sensor is highly selective for detecting low concentrations of NO_2_. The reason for the highest response enhancement to NO_2_ is not clear yet, but this outcome is very interesting with regards to fabricating a highly selective NO_2_ sensor. When a sensor is applied to an actual environment and exposed to a wide range of pollutants and VOCs, the PS/WO_3_ NRs–Pd NPs sensor can discriminate NO_2_ in a few seconds among several analytes in a mixed gas.

### 3.3. NO_2_-Sensing Mechanism

**Figure 8:**
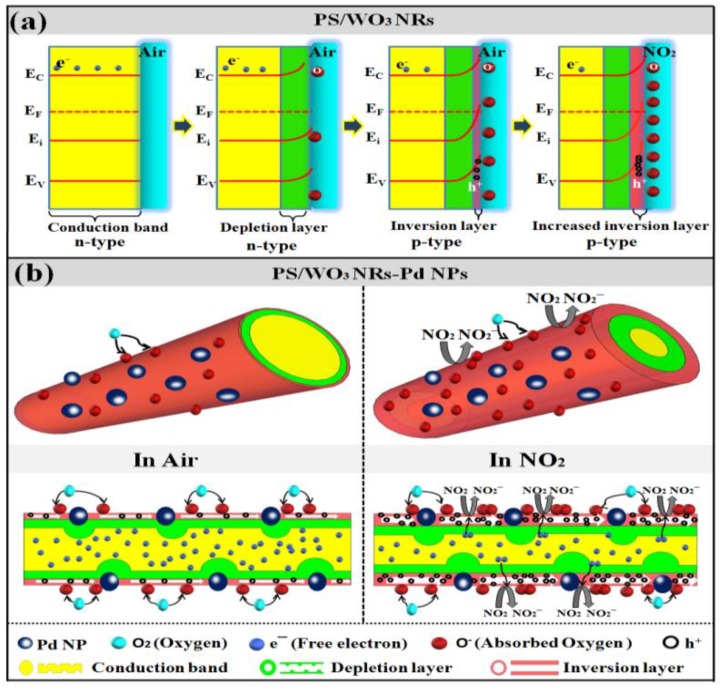
(**a**) Schematic energy-level diagram of the PS/WO_3_ NRs sensor: from n-type semiconductor with flat band structure to n-type semiconductor with depletion layer, p-type semiconductor with inversion layer, and p-type semiconductor with increased inversion layer; (**b**) schematic adsorption-reaction gas-sensing mechanism of PS/WO_3_ NRs–Pd NPs sensor: in air and in NO_2_.

The gas-sensing mechanism can be explained by the absorption-reaction mechanism. Figure 8a shows the energy-level diagram of the PS/WO_3_ NRs sensor, starting from n-type semiconductor with flat band structure to n-type semiconductor with depletion layer, p-type semiconductor with inversion layer, and p-type semiconductor with increased inversion layer. When the PS/WO_3_ NRs sensor is exposed to the air atmosphere, the oxygen molecules adsorb on the surface of WO_3_ NRs, which capture the free electrons from conduction band of WO_3_ NRs and form chemisorbed ion oxygen species (O_2_^−^, O^−^, and O^2−^). Both PS and WO_3_ NRs can absorb a large number of surface oxygen molecules due to their specific nanostructure, resulting in a narrower width of the conduction band due to capturing more free electrons, thereby increasing the width of the depletion region. As the free electrons are further captured, the depletion region increases and an inversion layer (the intrinsic Fermi level E_i_ lies above the Fermi level E_F_) appears and replaces the depletion layer, which means that holes become main charge carriers in the inversion layer and indicates that the conduction type of WO_3_ NR_S_ transforms from n-type to p-type. When the PS/WO_3_ NRs sensor is exposed to NO_2_, the NO_2_ molecules trap more free electrons, resulting in increasing the concentration of the holes in the inversion layer. The reaction kinematics are shown in Equations (2)–(6) [43]:

O_2(gas)_ → O_2(ads)_ (2)

O_2(ads)_ + e^−^ → O_2(ads)_^−^ (3)

O_2(ads)_^−^ + e^−^ → 2O_(ads)_^−^ (4)

NO_2(gas)_ + e^−^ → NO_2(ads)_^−^ (5)

NO_2(gas)_ + O_2(ads)_^−^ + 2e^−^ → NO_2(ads)_^−^ + 2O_(ads)_^−^ (6)

For enhanced NO_2_-sensing performance with decoration of Pd NPs, the following mechanism can be proposed. Figure 8b illustrates the adsorption-reaction gas-sensing mechanism of PS/WO_3_–Pd NPs in the air atmosphere and in an NO_2_ atmosphere. The decoration of Pd NPs decreases the width of the conduction band of WO_3_ NRs via depletion layer modulation at the WO_3_–Pd contact interfaces [44]. Because the work function of Pd (5.5 eV) is bigger than that of WO_3_ (4.8 eV), when Pd NPs have contact with WO_3_ NRs, electrons flow from the WO_3_ NRs to the Pd NPs and a potential barrier forms between WO_3_ NRs and Pd NPs. Upon exposure to NO_2_, many more free electrons are captured from WO_3_ NRs because of the contact between WO_3_ NRs and Pd NPs, resulting in a higher concentration of holes in the depletion layer. This leads to a further reduction of resistance and an increase in the sensor response. In addition, according to the spillover effect [19,20,44], the catalytic activity of Pd NPs accelerates the dissociation of oxygen molecules on the surface of WO_3_ NRs and causes a spillover of the chemisorbed ion oxygen species. More chemisorbed ion oxygen species on the surface of WO_3_ NRs provides more sensing sites, hence an enhanced gas sensor response. However, because too many Pd NPs cover the surface of WO_3_ NRs with increased Pd decoration, the contact area between WO_3_ NRs and oxygen molecules decreases, leading to less adsorption of oxygen species on the surface of WO_3_ NRs and less response enhancement, despite of the spillover effect of Pd NPs.

## 4. Conclusions

PS/WO_3_ NRs–Pd NPs were successfully synthesized by thermal oxidizing W film on the PS substrate and decorating Pd NPs on the surface of WO_3_ NRs by in-situ reduction of the Pd complex solution. Results demonstrated that decorating with Pd NPs and combining with PS substrate have a great influence on enhancing the gas-sensing performance of the WO_3_ NRs gas sensor. Compared with the PS/WO_3_ NRs gas sensor and the Si/WO_3_ NRs–Pd NPs gas sensor, the PS/WO_3_ NRs–Pd NPs gas sensor exhibited a higher and quicker response and better selectivity to NO_2_ due to its extremely high surface area to volume ratio and spillover effect. Moreover, the PS/WO_3_ NRs–Pd NPs gas sensor can detect NO_2_ as low as 0.25 ppm at RT, which makes it compatible with conventional silicon microfabrication technologies. Thus, it is promising as a NO_2_ sensor with low power consumption and excellent selectivity.

## Figures and Tables

**Table 1:** Comparisons of NO_2_ concentration, response, and working temperature of the PS/WO_3_–Pd60 sensor with those of other nanomaterial sensors.

Nanomaterials	NO_2_ Concentration (ppm)	Response	Working Temperature (°C)	References
PS/WO_3_–Pd60	2	5.2	RT	present work
WO_3_ film–Pd	10	0.42	200	[3]
Rosemine–SiO_2_/TiO_2_ composite	50	84%	RT	[4]
TiO_2_ NPs attached CuO NWs	10	5	300	[8]
Pd-loaded In_2_O_3_ nanowires	5	4.05	110	[15]
WO_3_ encapsulated with ZnO	5	2.8	300	[16]
